# A minimally invasive, lentiviral based method for the rapid and sustained genetic manipulation of renal tubules

**DOI:** 10.1038/srep11061

**Published:** 2015-06-05

**Authors:** Judit Espana-Agusti, David A. Tuveson, David J. Adams, Athena Matakidou

**Affiliations:** 1 Department of Oncology, University of Cambridge, CRUK Cambridge institute, Cambridge, CB2 0RE, UK; 2 Cold Spring Harbor Laboratory, 1 Bungtown Road, Cold Spring Harbor, NY 11724, USA; 3 Experimental Cancer Genetics, Wellcome Trust Sanger Institute, Hinxton, CB10 1SA, UK

## Abstract

The accelerated discovery of disease-related genes emerging from genomic studies has strained the capacity of traditional genetically engineered mouse models (GEMMs) to provide *in-vivo* validation. Direct, somatic, genetic engineering approaches allow for accelerated and flexible genetic manipulation and represent an attractive alternative to GEMMs. In this study we investigated the feasibility, safety and efficiency of a minimally invasive, lentiviral based approach for the sustained *in-vivo* modification of renal tubular epithelial cells. Using ultrasound guidance, reporter vectors were directly injected into the mouse renal parenchyma. We observed transgene expression confined to the renal cortex (specifically proximal and distal tubules) and sustained beyond 2 months post injection. Furthermore, we demonstrate the ability of this methodology to induce long-term, *in-vivo* knockdown of candidate genes either through somatic recombination of floxed alleles or by direct delivery of specific shRNA sequences. This study demonstrates that ultrasound-guided injection of lentiviral vectors provides a safe and efficient method for the genetic manipulation of renal tubules, representing a quick and versatile alternative to GEMMs for the functional characterisation of disease-related genes.

Though genetically engineered mouse models (GEMMs) have been instrumental in modelling human disease for biological studies, they can be expensive, time-consuming and difficult to generate, especially when investigation of complex traits, like cancer, is required. Non-germline approaches allow for accelerated and flexible genetic manipulation of models permitting the study of multiple genes or gene combinations, and high-throughput functional genetic screens within the appropriate cell/tissue context^1^. Lentiviral vectors represent an appealing tool for such approaches because of their ability to stably incorporate into the genome with high efficiency and have been successfully applied for the somatic genetic modification of a number of tissues^2^,^3^,^4^. Lentiviral vector delivery into the mouse kidney has been limited by the requirement for invasive surgical approaches involving exposure of the kidney and injection of viral preparations under direct visualisation^5^,^6^,^7^. Such approaches are technically demanding, time consuming and carry significant morbidity and mortality. Furthermore, studies to-date have examined only short time-points (days to weeks) following transduction. Approaches that allow long-term genetic modifications are essential for the *in-vivo* modelling of renal disease.

In this study we report the development and validation of a novel minimally invasive method for the *in-vivo* sustained genetic manipulation of the mouse renal tubular epithelium using lentiviral vectors and establish the feasibility of this approach as an alternative to traditional germline models.

## Results

### Feasibility and safety of direct renal intraparenchymal delivery of lentiviral vectors using a minimally invasive, ultrasound-guided approach

**Figure 1:**
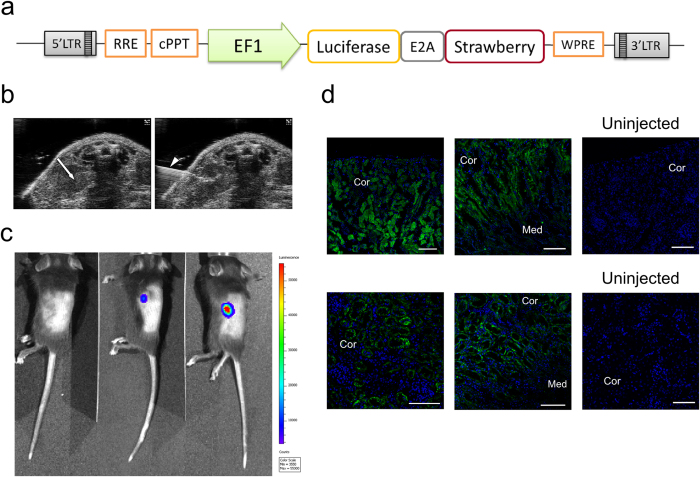
Efficient and sustained renal tubular gene delivery via Ultrasound-guided intraparenchymal injection of lentiviral vectors. (**a**) Diagram of pELS lentiviral construct. LTR, long terminal repeat; RRE, Rev response element; cPPT, central polypurine tract; EF1, elongation factor 1 alpha promoter; E2A, self-cleaving 2A peptide; WPRE, Woodchuck Hepatitis Virus Posttranscriptional Regulatory Element. (**b**) Representative ultrasound images of left renal parenchymal injections in adult mice. White arrow shows kidney location, arrow tip points to the injection needle. (**c**) Whole body bioluminescence of kidney-specific luciferase expression in representative C57/BL6J mice that underwent ultrasound-guided left intrarenal injection of either ELS or control (no luciferase) lentiviral vector (left hand mouse) at 7 days post injection. The relative luminescence intensity is indicated by a colour scale bar. (**d**) Representative immunofluorescent images of renal sections of adult (top panels) and neonatal (bottom panels) ELS injected and control (uninjected) kidneys, 60 days post infection. Strawberry expression was limited to the renal cortex and corticomedullary junction. Cor, cortex; Med, medulla. Scale bars, 50 μm.

To determine whether lentiviral vectors could be safely and efficiently delivered using a non-surgical approach we utilised a reporter third generation vector in which the expression of Luciferase and Strawberry fluorescent protein is driven by the constitutive EF1a promoter (ELS lentiviral vector, Fig. 1a). We performed single, low volume (10 μl) ultrasound (US) guided microinjection into the left renal parenchyma of adult (8 week, n = 5) and neonatal (7-12 day, n = 5) C57BL/6 mice (Fig. 1b). Each procedure lasted a maximum of 15 minutes and all injected mice recovered well post anaesthesia with no adverse events observed. Histopathological examination of both adult and neonatal kidneys at 7, 15 and 60 days post transduction did not reveal any morphological alterations or infiltrations within the renal parenchyma suggesting the absence of a persistent inflammatory response secondary to lentiviral infection.

To establish lentiviral vector integration and transgene expression *in-vivo* bioluminescence was assessed at 7 days post US-guided intraparenchymal injection. Luminescence was successfully detected from the left flank of ELS injected mice but not from litter-mates injected with a control (no luciferase) lentiviral vector (Fig. 1c). Furthermore, immunohistochemical analysis of injected kidneys at 7, 15 and 60 days post infection revealed sustained Strawberry expression limited to the renal cortex and corticomedullary junction (Fig. 1d), supporting stable lentiviral integration and transgene expression.

### Cell and tissue specificity of lentiviral transduction

**Figure 2:**
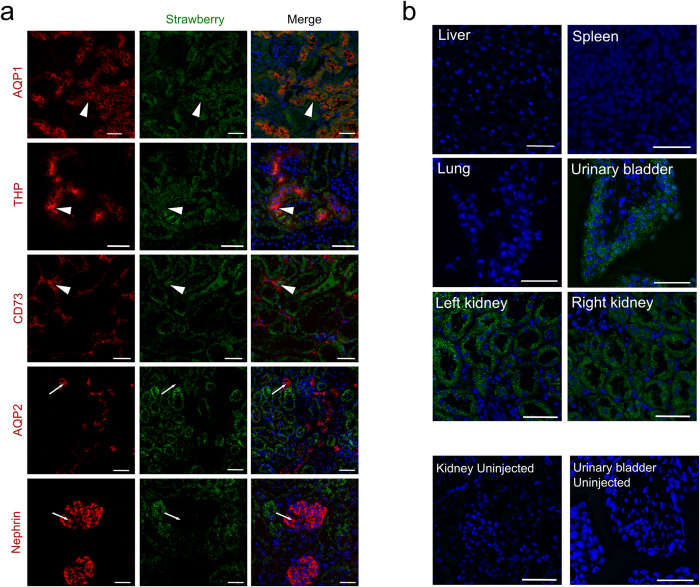
Intraparenchymal delivery of lentiviral vectors preferentially infects epithelial cells of both kidneys and urinary bladder. (**a**) Confocal images of Strawberry expression in renal sections of ELS infected kidneys 60 days post injection. Sections were stained with the indicated antibodies and merged images with DAPI (blue) are presented. Preferential transduction was observed in proximal (AQP1) and distal (THP) renal tubular epithelial cells and renal fibroblasts (CD73) (white arrowheads). Strawberry was not localised to collecting ducts (AQP2) or podocytes (Nephrin) (white arrows). Scale bars, 50 μm. (**b**) Confocal images of Strawberry expression in the liver, spleen, lung, urinary bladder and left and right kidney of ELS infected animals 60 days post left intrarenal injection (top panels) and uninjected control animals (bottom panels). Sections were stained with an antibody against Strawberry (green) and merged images with DAPI (blue) are presented. Scale bars, 50 μm.

Cell type and tissue specificity of transduction was determined both at 15 and 60 days post injection. Co-localisation studies revealed preferential infection of mainly proximal renal tubular epithelial cells, with lesser infection rates detected in distal tubules and fibroblasts (Fig. 2a). Collecting ducts and podocytes were spared (Fig. 2a). Though no reporter expression could be detected in the liver, spleen or lungs of injected animals, Strawberry expression was seen in the contralateral (right) kidney and in the epithelium of the urinary bladder (Fig. 2b), suggesting the occurrence of trans-ureteric but not haematogenous viral spread. To evaluate the extent of renal lentiviral transduction achieved by our approach, we examined multiple sections of both left and right kidneys of injected mice. Widespread cortical expression of Strawberry was observed at multiple levels throughout both kidneys further supporting lentiviral diffusion within the kidneys most likely through the ureteric system.

The age of mice at the time of the procedure did not influence the observed level, extent, cell or tissue specificity of lentiviral transduction.

### Direct delivery of Cre-recombinase and RNAi for the sustained genetic modification of renal tubular epithelial cells

We next sought to investigate the potential application of this approach as an alternative to traditional germline mouse models of kidney disease. As our specialist interest lies in renal cancer research we chose to apply our methodology for the modification of genes known to be deleted in human renal cancer (*VHL* and *TSC1*).

**Figure 3:**
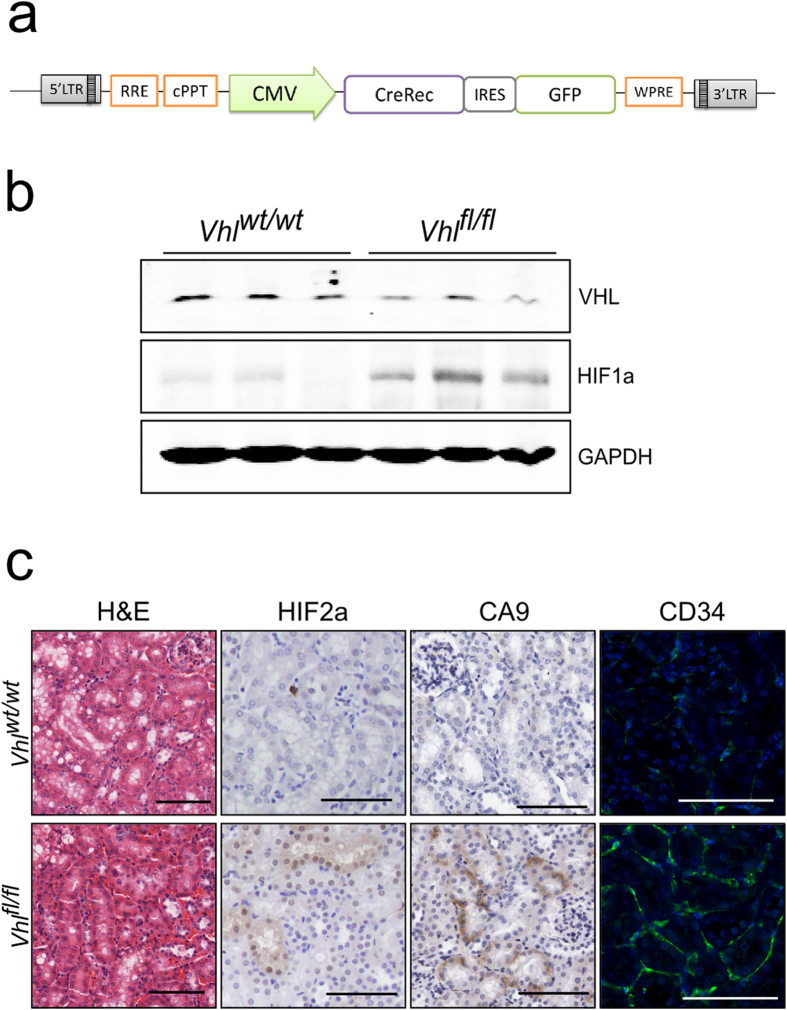
*In-vivo* lentiviral delivery of Cre-recombinase to renal tubular epithelium results in recombination of target genes. (**a**) Diagram of pCCIE lentiviral construct. Cre, Cre-recombinase; IRES, internal ribosome entry site; GFP, Green fluorescent protein. (**b**) Anti-VHL, HIF1a and GAPDH immunoblots of renal cortical protein lysates from *Vhl*^*wt/wt*^ and *Vhl*^*fl/fl*^ mice intrarenally injected with CCIE. Samples were collected 12 months post infection with each column representing an individual mouse. Blots were cropped to improve clarity, full-length blots are presented in Supplementary Fig. S3a. (**c**) Histological images of renal sections from *Vhl*^*wt/wt*^ and *Vhl*^*fl/fl*^ mice intrarenally injected with CCIE at 12 months post injection (stains and antibodies as indicated). Scale bars, 100 μm.

We first examined the feasibility and efficiency of directly delivering Cre-recombinase and inducing conditional deletion of *Vhl* within renal tubular epithelial cells of GEMM, thereby bypassing the need for tissue specific germline Cre-alleles. We generated the lentiviral vector CCIE, in which the cytomegalovirus promoter drives Cre-recombinase and the reporter GFP (Fig. 3a) and performed intrarenal injections in cohorts of mice homozygous or wild-type for the *loxP*-flanked *Vhl* allele^8^ (n = 21 and 4, respectively). Mice were analysed at 6, 12 and 16 months post-injection. Recombination of *Vhl* in kidneys of infected Vhl^fl/fl^ mice and subsequent reduction in VHL protein levels within renal cortical lysates was confirmed by immunoblot (Fig. 3b). *Vhl* deleted kidneys were of normal external appearance and parenchymal mass, and did not display histological abnormalities in the structure of the renal tubules (Fig. 3c) consistent with previously published human and mouse data^9^,^10^,^11^. Furthermore, as seen in germline mouse models of biallelic renal *Vhl* deletion we observed accumulation of HIF1a (Fig. 3b), HIF2a and their downstream target CA9 (Fig. 3c)^10^,^11^, as well as an increase in cortical vascularisation (CD34 immunofluorescence, Fig. 3c)^12^. These results support the use of lentiviral intra-parenchymal delivery of Cre-recombinase as an alternative system for fast and efficient genetic modification of renal tubular epithelial cells.

**Figure 4:**
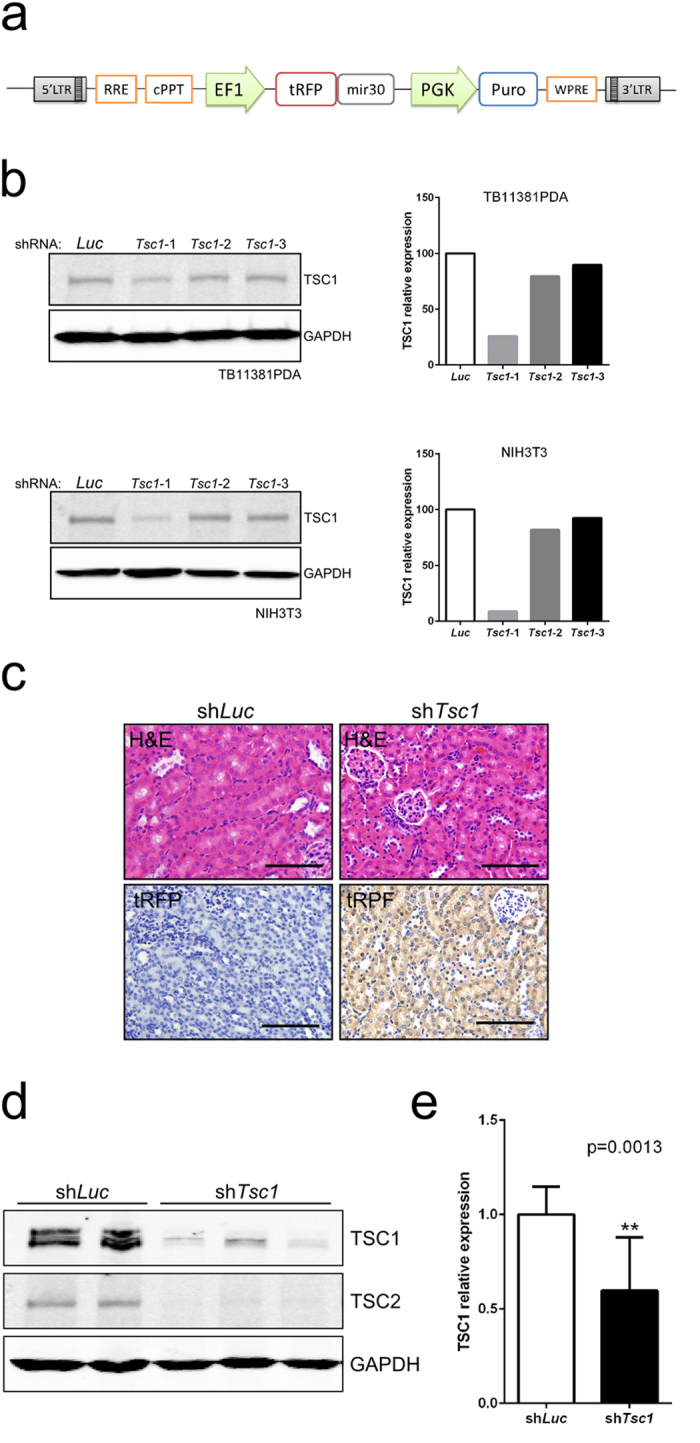
Efficient renal tubule-specific TSC1 knockdown using intrarenal lentiviral shRNA *in-vivo*. (**a**) Diagram of pERP lentiviral construct. EF1, elongation factor 1 alpha promoter; tRFP; turbo Red fluorescent protein; mir30, mir30-shRNA sequence; PGK, phosphoglycerate kinase I promoter; Puro, puromycin. (**b**) Anti-TSC1 and GAPDH immunoblots (left panels) and corresponding relative quantification of TSC1 protein levels (right panels) of protein lysates of TB11381PDA (mouse pancreatic adenocarcinoma) and NIH3T3 cells infected with ERP harbouring Tsc1-specific shRNAs (shTsc1-1, shTsc1-2 and shTsc1-3) or control luciferase-specific shRNA (shLuc). Blots were cropped to improve clarity, full-length blots are presented in Supplementary Fig. S3b. (**c**) Representative histological images of renal sections from ERP-shTsc1-1 and ERP-shLuc infected animals 12 months post left intrarenal injection and uninjected control animals (stains and antibodies as indicated). Scale bars, 100 μm. (**d**) Anti-TSC1, TSC2 and GAPDH immunoblots of renal cortical protein lysates from ERP-shTsc1-1 and ERP-shLuc intrarenally infected mice. Samples were collected 12 months post infection with each column representing an individual mouse. Blots were cropped to improve clarity, full-length blots are presented in Supplementary Fig. S3c. (**e**) Relative quantification of TSC1 protein levels from renal cortical lysates of ERP-shTsc1-1 (n = 7) and ERP-shLuc (n = 4) infected mice at 12 months post injection. Data represent mean ± s.d. Differences between groups were assayed using Student’s *t*-test.

Lentiviral systems also provide a safe and rapid approach for RNAi-mediated loss of function studies. As a proof of principle, we generated the lentiviral vector ERP, in which the reporter turbo-RFP and a mir30-shRNA sequence are driven by the EF1a promoter (Fig. 4a). Three shRNA sequences targeting the mouse *Tsc1* gene, known to be mutated in renal cancer (shTsc1-1, shTsc1-2 and shTsc1-3) and an shRNA targeting luciferase (shLuc; control)^13^, were selected for further validation. Efficiency of TSC1 knockdown was examined *in-vitro* in NIH3T3 and TB11381PDA cells (a mouse pancreatic adenocarcinoma cell line); lentiviral infection with shTsc1-1 displayed the most efficient knockdown (~80%) and was selected for subsequent *in-vivo* experiments (Fig. 4b).

Five week old C57BL/6J mice underwent US-guided left intra-renal injection with ERP lentiviruses carrying either shTsc1-1 or shLuc (n = 10 and n = 5 respectively). Mice were analysed at 4 and 12 months post infection. No macroscopic or microscopic histological abnormalities could be detected in the kidneys of infected animals (Fig. 4c). Lentiviral transduction was confirmed by immunohistochemistry against turbo-RFP (Fig. 4c). TSC1 protein levels in the cortex of kidneys infected with the ERP-shTsc1-1 virus were significantly reduced one year following lentiviral delivery (Fig. 4d,e). We observed a corresponding reduction in TSC2 levels (Fig. 4d) consistent with the role of TSC1 in stabilising TSC2 protein levels^14^. The TSC complex is a key negative regulator of the mTORC1 complex^15^. Both *in-vivo* and *in-vitro* models have demonstrated that complete (biallelic) loss of TSC1 leads to the activation of mTORC1 and its downstream effectors (e.g. S6 ribosomal protein)^16^,^17^,^18^. To investigate the ability of the shTSC1-1 hairpin to induce complete TSC1 knockdown we performed immunohistochemical analyses of phospho-S6. No differences in renal phospho-S6 expression were detected between ERP-shTsc1-1 and ERP-shLuc injected animals (supplementary Fig. S1), suggesting the persistence of residual TSC1 protein within cells. Our results support the use of lentiviral vectors for safely and efficiently delivering shRNAs to the renal tubular epithelium resulting in the long term suppression of target proteins.

## Discussion

In this study, we demonstrate that US-guided intraparenchymal delivery of lentiviral vectors can induce sustained genetic modification of renal tubular epithelial cells bypassing the need to engineer novel GEMMs and to breed multiple animals for the generation of compound mutants. A big advantage of our approach is its safety and efficacy. The use of US-guidance allows lentiviral delivery using a minimally invasive procedure with no consequent mouse morbidity or mortality. Furthermore, viral transduction was achieved in both neonatal and adult kidneys, allowing genetic manipulation of both developing and mature tubular systems. As with previously published studies, viral transduction was limited to the cortical tubular epithelium with no infected cells seen in the glomeruli or medulla^5^,^6^,^7^. We also observed infection of urinary bladder epithelium and of the contralateral (uninjected) kidney but no infection within the liver, spleen or lungs of injected animals. These data support a model of transureteric viral spread which combined with the use of broad tropism lentiviral vectors (VSV-G pseudotyped) may have further enhanced the extent of transduction achieved by our model. Contralateral kidney transduction has been previously reported^6^, though of much lesser extent. This is most likely attributed to technical differences, as previous studies occluded the renal pedicle, essentially restricting the viral load to the injected kidney.

To our knowledge, this is the first study demonstrating sustained transgene expression at one year following renal lentiviral infection making it an attractive alternative to GEMMs for the study of chronic renal diseases and long-term outcomes. Using this approach we have successfully delivered Cre-recombinase and induced knockout of the *Vhl* gene in *Vhl*^*fl/fl*^ mice reproducing the phenotype observed in GEMMs. Furthermore, delivery of shRNA sequences targeting *Tsc1* resulted in sustained reduction of protein levels up to one year post infection. Though no phenotype was observed, this is likely to reflect the incomplete knockdown of TSC1 achieved by our shRNA sequence, most closely resembling *in-vivo* models of heterozygous *Tsc1* loss. GEMMs with constitutive heterozygous loss of *Tsc1* develop renal cysts and lesions from approximately 12 months with most pathology being detected in 15-18 month old mice^16^,^17^,^18^. Longer follow-up or younger age at time of injection is likely to have allowed the development of pathology in our model. Alternatively, improvements in the efficacy of Tsc1 gene knockdown either by increasing the amount of delivered transgene (higher injected viral load or repeated viral injections) or by selecting alternative targeting approaches (like the recently described vectors mediating CRISPR-Cas9 knockout of single or multiple genes^19^), could provide more robust and specific gene targeting.

The current sequencing efforts have greatly expanded our knowledge of the genetic landscape of human renal disease. Our proposed strategy of intraparenchymal lentiviral transduction offers flexibility and speed, allowing for rapid testing and functional validation of candidate renal tubular disease-causing variants and the elucidation of renal disease pathobiology.

## Methods

### Vectors and lentivirus production, purification and concentration

Constructs were cloned into the third-generation pBOBI lentiviral system that contains VSV-G and WPRE sequences for improved tropism and transgene expression (vector pTomo kindly donated by Prof Inder Verma, Salk Institute^3^). The pELS lentiviral vector was kindly donated by Dr Scott Lyons, University of Cambridge, UK. Maps of all vectors used are presented in supplemental Fig. S2. Briefly, to generate pCCIE, a Cre-recombinase fragment from the SV40CreERT2 vector^20^ and the CMV promoter and IRES-EGFP fragments of pTOMO were PCR amplified and inserted between the EcoRI and NotI (Cre-recombinase), PacI (CMV) and NotI (IRES-EGFP) sites of the pELS vector. To construct pERP, we amplified the tRFP-mir30 fragment of the pTRIPZ vector (Dharmacon) and inserted it into the NotI site of the pELS vector. Finally, a PGK-Puro cassette (kindly donated by Dr Scott Lyons, University of Cambridge) was inserted into the NheI site of the above vector. Target shRNA sequences were selected from published and on-line resources (supplemental Table S1) and cloned between the PspXI and EcoRI sites of the pERP vector.

VSV-G-pseudotyped lentiviral particles were produced as described previously^21^. All viral supernatants were concentrated approximately 100-fold by ultracentrifugation at 25,000 × g for 2 h. Lentiviral titres were established by infection of HEK293T cells with serially diluted virus and flow cytometry for fluorescence detection. We routinely produced 1–5 × 10^8^ infectious units/ml.

### *In-vivo* intraparenchymal vector injection

All animal work was carried out in accordance to Home Office UK regulations and the Animals (Scientific Procedures) Act 1986. All experimental protocols were approved by the Animal Welfare and Ethical Review Body (AWERB) of the University of Cambridge CRUK Cambridge Institute. We used the following mice: C57/BL6J (Charles River laboratories), B6.129S4(C)-*Vhl*^*tm1Jae*^/J (Jackson Laboratories). The Vevo 2100 (Visualsonics) with microinjection system was used for all procedures. Mice aged 7 days to 8 weeks old were anaesthetised with isofluorane and positioned prone on the ultrasound platform. Under high-resolution ultrasound guidance a bolus injection of 10 μl viral solution was microinjected into the left renal cortex using a 30G needle and a 50 μl glass Hamilton syringe.

### *In-vivo* bioluminescence imaging

Mice were anaesthetized with isofluorane and injected intraperitoneally with 10 μl per g of body weight d-luciferin (15 mg/ml; Perkin-Elmer). Bioluminescence images, each typically 3 min, large binning, f/stop 1, were then taken 10–20 min post-luciferin injection, using an IVIS 200 series imaging system (Perkin-Elmer).

### Histology and immunohistochemistry

At the experimental end-point animals were euthanized and tissues were fresh frozen in liquid nitrogen or fixed in 4% paraformaldehyde in PBS overnight. To review histology, slides were stained by haematoxylin and eosin (H&E). Immunohistochemistry (IHC) and immunofluorescence (IF) were performed using standard protocols. Specificity of immunostaining was assessed by incubation in the absence of primary antibody. We used the following primary antibodies: RFP for Strawberry detection (ab34771, 1:400; Abcam), Aquaporin 1 (NB-600–749, 1:500; Novus Biologicals), THP (AF5175, 1:100; R&D), Aquaporin 2 (ab105171, 1:1000; Abcam), Nephrin (AF3159, 1:100; R&D), CD34 (ab8158, 1:50; Abcam), CD73 (AF4488, 1:100; R&D), GFP (ab290, 1:1000; Abcam), HIF2a (NB100-132, 1:150; Novus Biologicals), CA9 (sc-25600, 1:200; Santa Cruz), turbo-RFP (AB234, 1:500; Evrogen) and pS6 (2211, 1:200; Cell Signalling). Secondary antibodies used were conjugated to HRP (IHC) or Alexa-fluor® fluorochromes (IF). Fluorescent images were obtained by confocal laser-scanning microscopy (Leica TCS SP5).

### Cell culture

HEK293T, NIH3T3 and TB11381PDA cells were cultured and maintained in Dulbecco’s modified Eagle’s medium supplemented with glucose and glutamine, with 10% FBS. For viral infections, NIH3T3 and TB11381PDA cells were plated in 96-well plates at 2,500 and 5,000 cells per well respectively and incubated with lentivirus overnight. After 8 days, we positively selected infected cells with puromycin (4 μg/ml) for 4 days and processed them for protein analyses.

### Immunoblotting

Cells or tissues were lysed in RIPA buffer with protease and phosphatase inhibitors and lysates were processed using standard methods. We used the following primary antibodies: GAPDH (ab9485, 1:2000; Abcam), VHL (sc-5575, 1:250; Santa Cruz), HIF1a (NB100-105, 1:500; Novus Biologicals), TSC1 (ab32936, 1:500; Abcam), TSC2 (4308, 1:1000; Cell Signalling). Secondary antibodies conjugated to IRDye 680 or 800 were used (Li-Cor). Fluorescent signals were quantified using the Odyssey Infrared Imaging System (Li-Cor).

### Statistics

Quantitative data are expressed as mean ± s.d. Differences between groups were assayed using Student’s *t*-test. Differences were considered significant when p < 0.05. Statistical manipulations were performed in GraphPad Prism (GraphPad Software, Inc).

## Additional Information

**How to cite this article**: Espana-Agusti, J. *et al.* A minimally invasive, lentiviral based method for the rapid and sustained genetic manipulation of renal tubules. *Sci. Rep.* **5**, 11061; doi: 10.1038/srep11061 (2015).

## Supplementary Material

Supplementary Information

## References

[b1] Heyer J., Kwong L. N., Lowe S. W. & Chin L. Non-germline genetically engineered mouse models for translational cancer research. Nat. Rev. Cancer 10, 470–480 (2010). 20574449 10.1038/nrc2877 PMC4602412

[b2] DuPage M., Dooley A. L. & Jacks T. Conditional mouse lung cancer models using adenoviral or lentiviral delivery of Cre recombinase. Nat. Protoc. 4, 1064–1072 (2009). 19561589 10.1038/nprot.2009.95 PMC2757265

[b3] Marumoto T. *et al.* Development of a novel mouse glioma model using lentiviral vectors. Nat. Med. 15, 110–116 (2009). 19122659 10.1038/nm.1863 PMC2671237

[b4] Friedmann-Morvinski D. *et al.* Dedifferentiation of neurons and astrocytes by oncogenes can induce gliomas in mice. Science 338, 1080–1084 (2013). 23087000 10.1126/science.1226929 PMC3595315

[b5] Gusella G. L., Fedorova E., Marras D., Klotman P. E. & Klotman M. E. *In vivo* gene transfer to kidney by lentiviral vector. Kidney Int. 61, S32–36 (2002). 11841609 10.1046/j.1523-1755.2002.0610s1032.x

[b6] Kim M. *et al.* Kidney-specific reconstitution of the A1 adenosine receptor in A1 adenosine receptor knockout mice reduces renal ischemia-reperfusion injury. Kidney Int. 75, 809–823 (2009). 19190680 10.1038/ki.2008.699 PMC2692553

[b7] Chen S. W. *et al.* Mice that overexpress human heat shock protein 27 have increased renal injury following ischemia reperfusion. Kidney Int. 75, 499–510 (2009). 19020532 10.1038/ki.2008.572 PMC2692047

[b8] Haase V. H., Glickman J. N., Socolovsky M. & Jaenisch R. Vascular tumors in livers with targeted inactivation of the von Hippel-Lindau tumor suppressor. Proc. Natl. Acad. Sci. U.S.A. 98, 1583–1588 (2001). 11171994 10.1073/pnas.98.4.1583 PMC29300

[b9] Mandriota S. J. *et al.* HIF activation identifies early lesions in VHL kidneys: evidence for site-specific tumor suppressor function in the nephron. Cancer Cell 1, 459–468 (2002). 12124175 10.1016/s1535-6108(02)00071-5

[b10] Frew I. J. *et al.* pVHL and PTEN tumour suppressor proteins cooperatively suppress kidney cyst formation. EMBO J. 27, 1747–1757 (2008). 18497742 10.1038/emboj.2008.96 PMC2435131

[b11] Mathia S. *et al.* Action of hypoxia-inducible factor in liver and kidney from mice with Pax8-rtTA-based deletion of von Hippel-Lindau protein. Acta Physiol. (Oxf.) 207, 565–576 (2013). 23384425 10.1111/apha.12058

[b12] Lee C. M. *et al.* VHL Type 2B gene mutation moderates HIF dosage *in vitro* and *in vivo*. Oncogene 28, 1694–1705 (2009). 19252526 10.1038/onc.2009.12 PMC2667565

[b13] Chang K., Elledge S. J. & Hannon G. J. Lessons from Nature: microRNA-based shRNA libraries. Nat. Methods 3, 707–714 (2006). 16929316 10.1038/nmeth923

[b14] Chong-Kopera H. *et al.* TSC1 stabilizes TSC2 by inhibiting the interaction between TSC2 and the HERC1 ubiquitin ligase. J. Biol. Chem. 281, 8313–8316 (2006). 16464865 10.1074/jbc.C500451200

[b15] Inoki K., Zhu T. & Guan K. L. TSC2 mediates cellular energy response to control cell growth and survival. Cell 115, 577–590 (2003). 14651849 10.1016/s0092-8674(03)00929-2

[b16] Kobayashi T. *et al.* A germ-line Tsc1 mutation causes tumor development and embryonic lethality that are similar, but not identical to, those caused by Tsc2 mutation in mice. Proc. Natl. Acad. Sci. U.S.A. 98, 8762–8767 (2001). 11438694 10.1073/pnas.151033798 PMC37509

[b17] Kwiatkowski D. J. *et al.* A mouse model of TSC1 reveals sex-dependent lethality from liver hemangiomas, and up-regulation of p70S6 kinase activity in Tsc1 null cells. Hum. Mol. Genet. 11, 525–534 (2002). 11875047 10.1093/hmg/11.5.525

[b18] Wilson C. *et al.* A mouse model of tuberous sclerosis 1 showing background specific early post-natal mortality and metastatic renal cell carcinoma. Hum. Mol. Genet. 14, 1839–1850 (2005). 15888477 10.1093/hmg/ddi190

[b19] Shalem O. *et al.* Genome-scale CRISPR-Cas9 knockout screening in human cells. Science 343, 84–87 (2014). 24336571 10.1126/science.1247005 PMC4089965

[b20] Feil R., Wagner J., Metzger D. & Chambon P. Regulation of Cre recombinase activity by mutated estrogen receptor ligand-binding domains. Biochem. Biophys. Res. Commun. 237, 752–757 (1997). 9299439 10.1006/bbrc.1997.7124

[b21] Tiscornia G., Singer O. & Verma I. M. Production and purification of lentiviral vectors. Nat. Protoc. 1, 241–245 (2006). 17406239 10.1038/nprot.2006.37

