# Supplementary material for: A minimally invasive, lentiviral based method for the rapid and sustained genetic manipulation of renal tubules
Source: Sci Rep. 2015 Jun 5;5:11061. doi: 10.1038/srep11061 (PMC4457145; doi:10.1038/srep11061)

SUPPLEMENTAL INFORMATION

A minimally invasive, lentiviral based method for the rapid and sustained genetic manipulation of renal tubules.

Judit Espana-Agusti [1], David A. Tuveson [1, 2], David J. Adams [3] and Athena Matakidou [1*]

[1] Department of Oncology, University of Cambridge, CRUK Cambridge institute, Cambridge, UK

[2] Cold Spring Harbor Laboratory, 1 Bungtown Road, Cold Spring Harbor, NY 11724, USA

[3] Experimental Cancer Genetics, Wellcome Trust Sanger Institute, Hinxton, UK

[*]Corresponding author:

Dr. Athena Matakidou, Department of Oncology, University of Cambridge CRUK Cambridge Institute, Li Ka Shing Centre, Robinson Way, Cambridge, UK, CB2 0RE.

Phone: 0044 1223 769527

Fax: 0044 1223 769510

Email: Athena.Matakidou@cruk.cam.ac.uk

TABLES

Table S1: Target sequences of mir30-shRNAs used for *in-vitro* and *in-vivo* lentiviral transduction.

Name	Target Sequence	Source
shTsc1-1	CGGAAGAAGCTGCAATATCTAA	HP_239722 (RNAi Codex)
shTsc1-2	AAAGAAAGAGCAGATTCCTCAA	V3LMM_450747 (Dharmacon)
shTsc1-3	CCGGGAGCTGTTCCGTAATAA	TRCN0000238186 (TRC Portal, Broad Institute)
shLuc	CCGCCTGAAGTCTCTGATTAA	Chang et al (2006), Nature Methods; 3(9): 707-714

SUPPLEMENTARY FIGURE LEGENDS

Supplemental Figure S1. Histological images of renal sections from animals intrarenally injected with ERP-shLuc or ERP-shTsc1 at 12 months post left intrarenal injection stained for phospho-S6. Scale bar 100µm.

Supplemental Figure S2. Maps of lentiviral vectors. (a) pELS, (b) pCCIE, and (c) pERP. CMV, cytomegalovirus promoter; LTR, long terminal repeat; RRE, Rev response element; cPPT, central polypurine tract; EF1, elongation factor 1 alpha promoter; E2A, self-cleaving 2A peptide; WPRE, Woodchuck Hepatitis Virus Posttranscriptional Regulatory Element; Cre, Cre-recombinase; IRES, internal ribosome entry site; GFP, Green fluorescent protein; tRFP, turbo Red fluorescent protein; mir30, mir30-shRNA sequence; PGK, phosphoglycerate kinase I promoter.

Supplemental Figure S3. Full length membranes of Western blots presented in figures 3 and 4 as captured using the Odyssey Infrared Imaging System (Li-Cor). (a) Full membranes of anti-VHL, HIF1a and GAPDH immunoblots of renal cortical protein lysates from $Vhl^{wt/wt}$ and $Vhl^{fl/fl}$ mice intrarenally injected with CCIE (figure 3b). Samples were collected 12 months post infection. Each column represents an individual mouse. (b) Full membranes of anti-TSC1 and GAPDH immunoblots of protein lysates of TB11381PDA (mouse pancreatic adenocarcinoma) and NIH3T3 cells infected with ERP harbouring Tsc1-specific shRNAs (shTsc1-1, shTsc1-2 and shTsc1-3) or control luciferase-specific shRNA (shLuc) (figure 4b). (c) Full membranes of anti-TSC1, TSC2 and GAPDH immunoblots of renal cortical protein lysates from ERP-shTsc1-1 and ERP-shLuc intrarenally infected mice (figure 4d). Samples were collected 12 months post infection. Each column represents an individual mouse.

SUPPLEMENTARY FIGURES

Supplemental figure S1

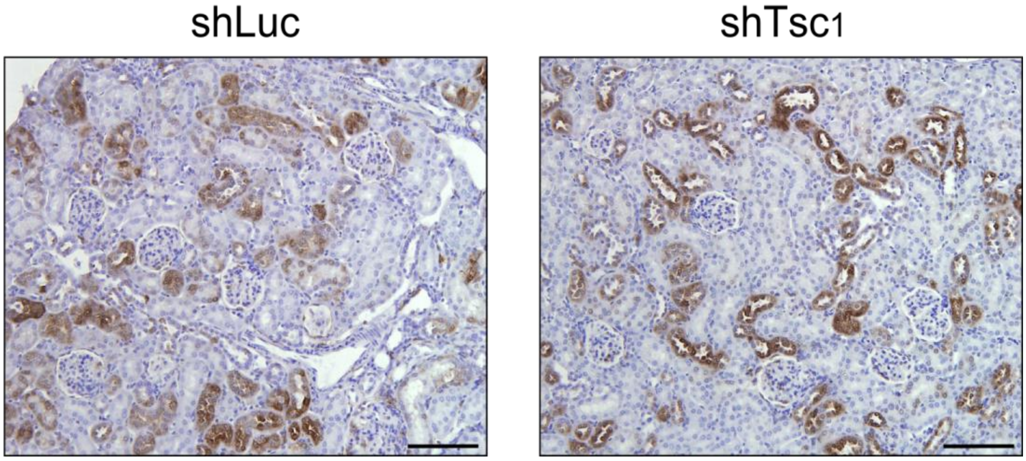

Supplemental figure S2

a

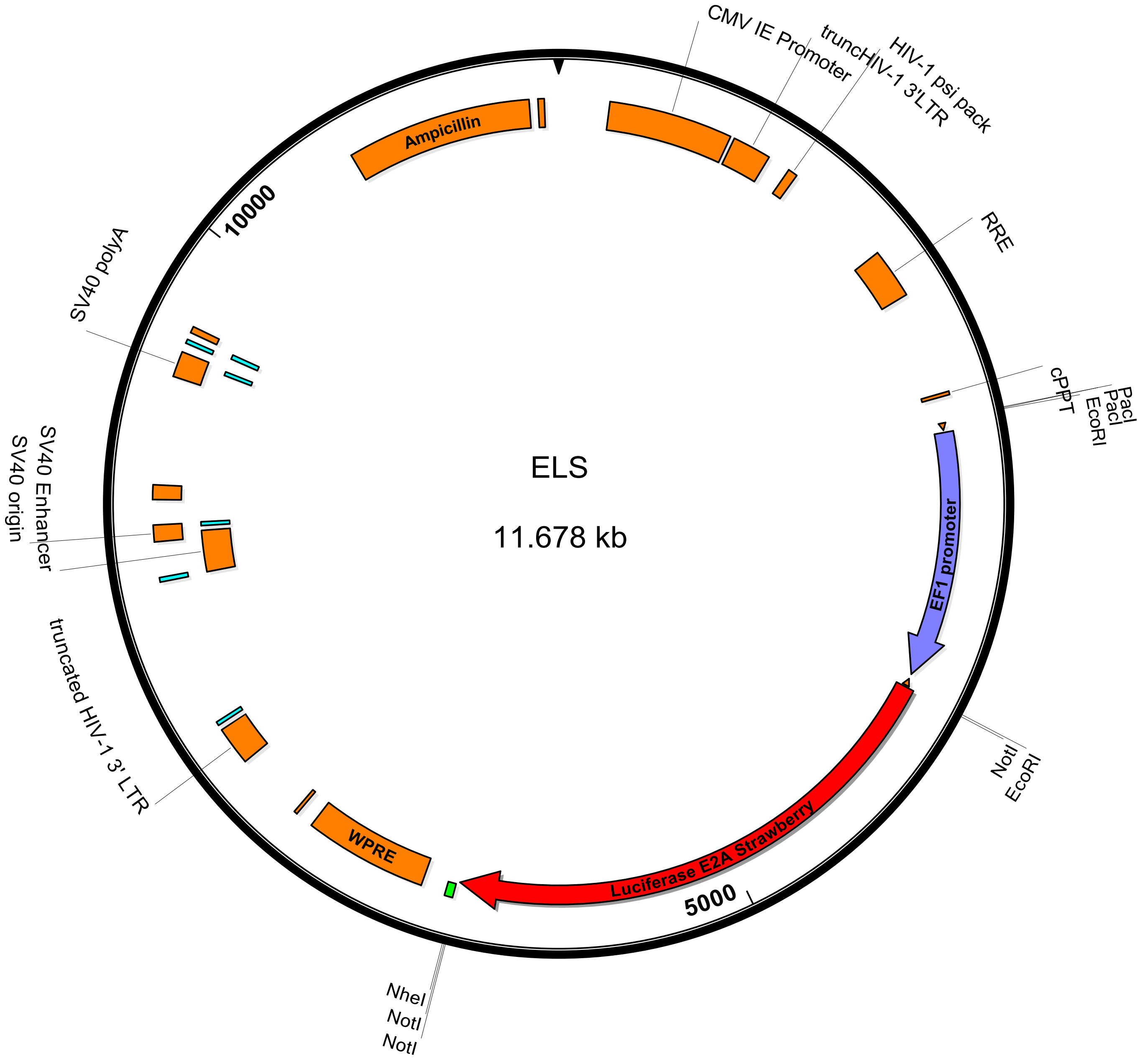

b

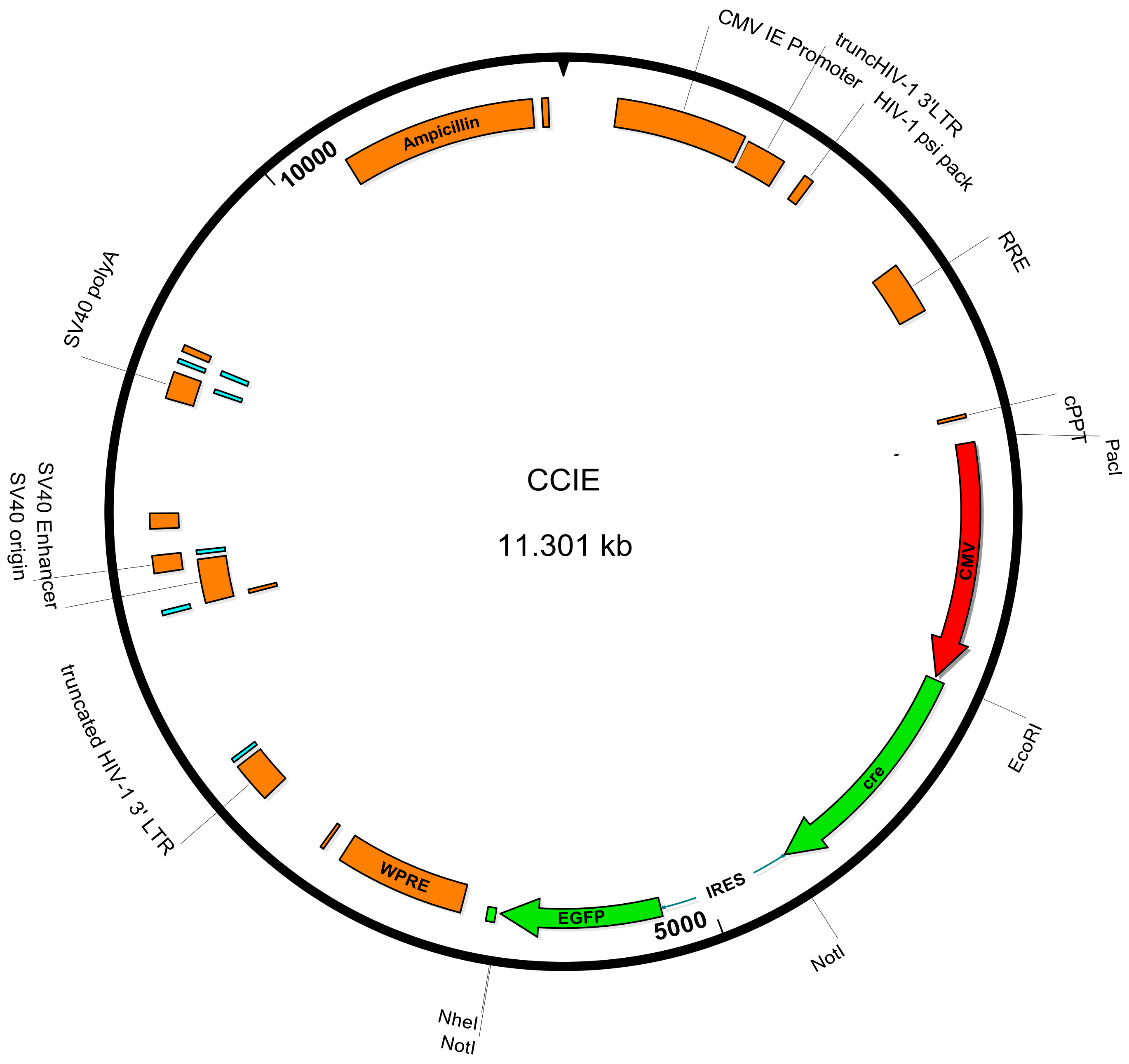

CCIE
11.301 kb
Ampicillin
10000
5000
CMV IE Promoter
truncHIV-1 3'LTR
HIV-1 psi pack
RRE
cPPT
PacI
CMV
EcoRI
cre
IRES
NotI
EGFP
NheI
NotI
WPRE
truncated HIV-1 3' LTR
SV40 Enhancer
SV40 origin
SV40 polyA

c

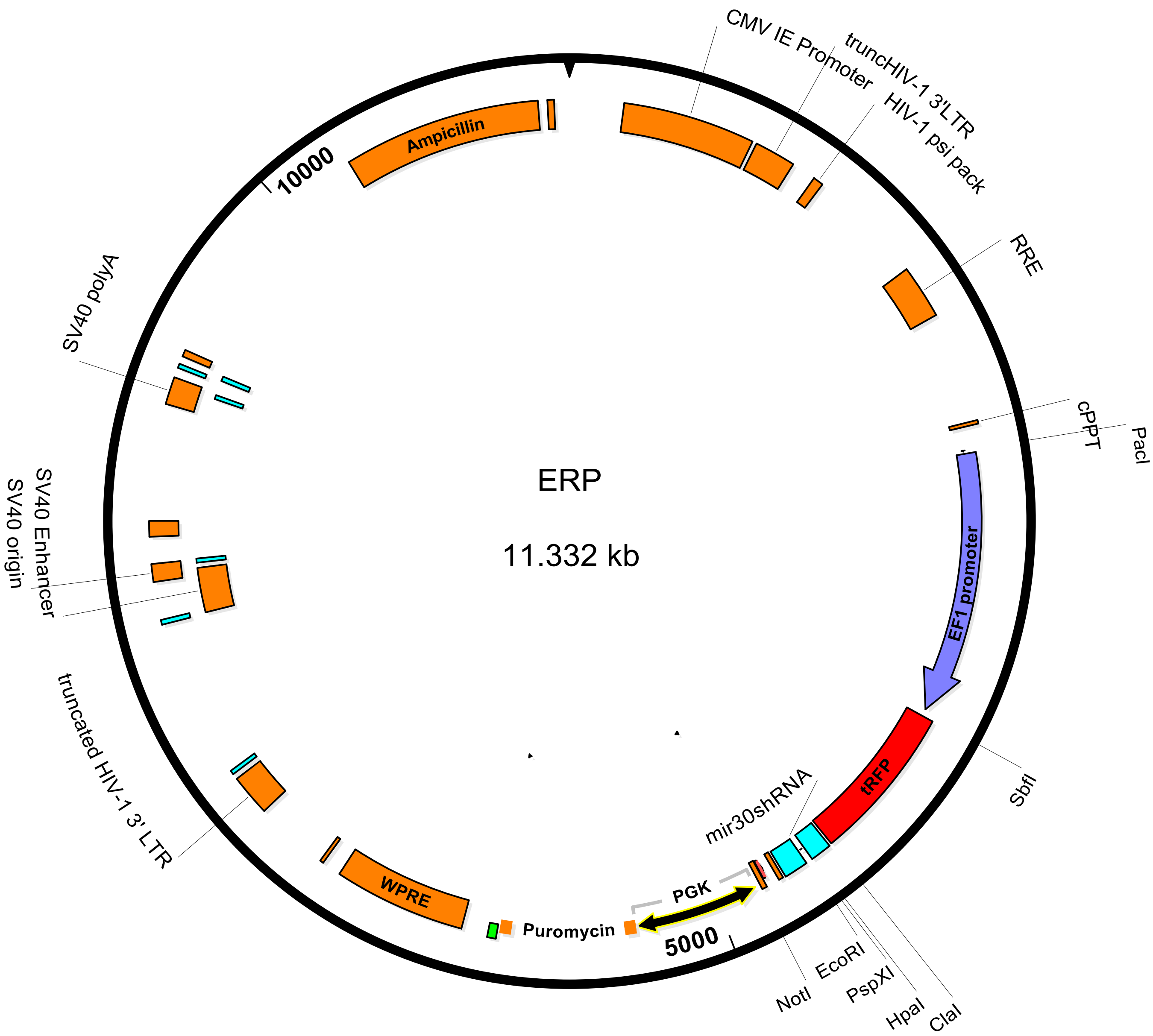
ERP
11.332 kb
CMV IE Promoter
truncHIV-1 3'LTR
HIV-1 psi pack
RRE
cPPT
PacI
EF1 promoter
tRFP
SbfI
mir30shRNA
ClaI
HpaI
PspXI
EcoRI
NotI
PGK
5000
Puromycin
WPRE
truncated HIV-1 3' LTR
SV40 origin
SV40 Enhancer
SV40 polyA
10000
Ampicillin

Supplemental figure S3

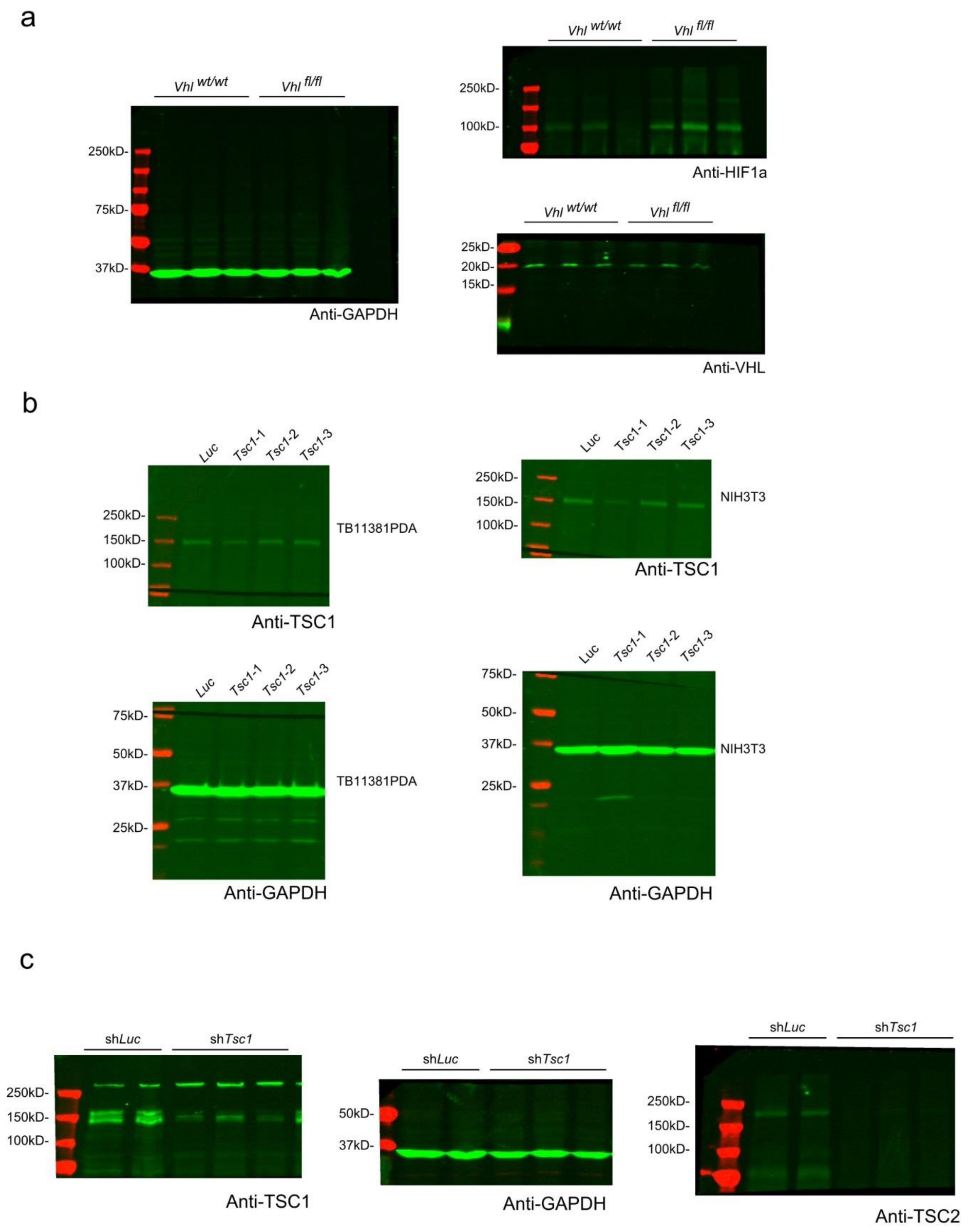

Supplement: Supplementary Information [file srep11061-s1.pdf]
